# Insecticide resistance comes of age

**DOI:** 10.1186/gb4162

**Published:** 2014-02-25

**Authors:** Richard H ffrench-Constant

**Affiliations:** 1 Biosciences, University of Exeter, Penryn Campus, Falmouth TR10 9EZ, UK

## Abstract

A new study integrates biochemistry, genetics and structural biology to reveal the mechanism of metabolic resistance in a vector mosquito in unprecedented detail.

See related research http://genomebiology.com/2014/15/2/R27

## The search for adaptations underlying insecticide resistance

Despite the apparent perfect storm of recent advances in genomics, transcriptomics and structural biology, insecticide resistance has been slow to embrace all of these new techniques as one enabling package. The field of resistance has therefore been effectively stuck in first gear, with the dissection of resistance mechanisms in non-model (non-*Drosophila*) insects requiring painstaking paper-by-paper dissection of the underlying genetics, biochemistry and, rarely, the structural biology of the associated metabolic enzyme or insecticide target [1]. However, an article in this issue of *Genome Biology* by Riveron and colleagues [2] looks to change all that, setting out a blistering series of experiments that unite genetics, biochemistry and structural biology to solve a long-standing question: which enzymes really can confer resistance to the organochloride dichlorodiphenyltrichloroethane (DDT) in insects?

Insects can evolve resistance to insecticides either by developing target-site insensitivity - changes in the receptors or enzymes that the drug targets - or by upregulating or altering the catalytic properties of detoxifying enzymes. These enzymes typically either perform a chemical attack on the insecticides before they reach their target - for example, esterases that can break crucial ester bonds and cytochrome P450 proteins that perform oxidative attack - or, in some cases, can also act as a sequestering sponge for the active insecticide. Ultimately, the insect needs both to degrade the insecticide into non-neuroactive components and also to promote its water solubility so that it can be more readily excreted. Enzymes known as glutathione S-transferases (GSTs), for example, promote water solubility by forming a complex between an insecticide and glutathione before excretion of the agent.

In the face of the large multigene families that encode this startling array of metabolic enzymes, the search for specific enzymes proven to be able to metabolize DDT, and indeed the search for resistance-associated point mutations, has taken on the feel of a search for the Holy Grail itself. Thus, point mutations associated with metabolic resistance have now been described in the alpha esterase E7 from the housefly [3] and carboxylesterase E3 from the Australian sheep blowfly [4] (in those cases to organophosphates rather than organochlorides), but both of these detailed studies have involved long, difficult and often controversial bodies of work that are only now beginning to integrate with the structural biology of the associated enzymes. Likewise, the involvement of GSTs in DDT metabolism itself has been historically one of the most debated subjects in the entire field [1].

## Genetics, biochemistry and structural biology

So which metabolic enzymes are involved in DDT resistance in the African malaria vector *Anopheles funestus* and how does this knowledge help us address the age-old issue regarding the role for GSTs in DDT metabolism? Furthermore, are these metabolic enzymes simply overexpressed (genetic upregulation of an enzyme with the same metabolic capabilities) or are they also better able to metabolize DDT (point mutations changing the catalytic properties of the encoded enzyme itself)? To address both these questions, Riveron and colleagues compared mosquitoes from Pahou, Benin, that could survive a discriminating dose of DDT with those that were either unexposed or from a susceptible laboratory strain. They looked at microarray data comparing resistant and susceptible insects and showed that a range of possible resistance genes were upregulated, including those encoding a candidate GST, two cytochrome P450 enzymes and a carboxylesterase. Genes upregulated in the microarray were then also confirmed to indeed be over-transcribed using quantitative RT-PCR. Through a process of elimination, it was argued that *GSTe2* plays the central role in DDT resistance in Benin. First, no knockdown resistance (*kdr*) mutation was detected in the gene encoding the voltage-gated sodium channel *paralytic* (*para*), which forms the actual target site for both DDT and pyrethroids. Second, although two different P450 genes were upregulated, it was thought that, based on earlier work, these were unable to metabolize DDT. GSTe2 therefore emerged as the clear candidate for the dominant resistance-associated enzyme in this strain.

To understand the experiments that the team next used to prove that GSTe2 was indeed responsible for resistance, it is perhaps best first to outline the formal possibilities. First, it is possible that simple overexpression of the wild-type GSTe2 enzyme alone might be sufficient to cause resistance - that is, that overexpression of the unaltered enzyme alone is sufficient to metabolize and/or sequester physiologically relevant levels of DDT. Second, it is also possible that catalytic changes in GSTe2 that increase its ability to deal with (sequester and/or metabolize) DDT could also alone result in resistance. Finally, of course, it is possible that resistance is associated both with genetic upregulation and also with changes in the catalytic properties of GSTe2 itself (presumably associated with amino acid replacements in the active site of the enzyme that improve docking with DDT and metabolism).

From the array data, it is clear that *GSTe2* is indeed overexpressed in the resistant strain, but is the enzyme mutated and does this mutation improve its ability to handle DDT? To address this question, the team used a number of approaches. First, they sequenced the open reading frame of the gene encoding GSTe2 from both susceptible and resistant strains and found that a mutation replacing a single leucine amino acid (L119 becoming F119) was consistently associated with resistance. This mutant (119 F) therefore instantly became a candidate resistance-associated mutation. Second, the authors measured directly the DDT dehydrochlorinase activity of the wild-type and mutant 119 F (resistant) enzyme. This showed that the resistant 119 F enzyme is 3.4 times more efficient at metabolizing DDT than the susceptible enzyme, as confirmed by the higher catalytic efficiency of the resistant enzyme for DDT (316.3 μM^-1^ s^-1^ versus 92.0 for the susceptible enzyme). Similarly, significant metabolism of the pyrethroid insecticide permethrin was also detected, suggesting the formal possibility that pre-selection with DDT could also have conferred subsequent GSTe2-mediated resistance to the pyrethroid insecticides designed to replace the use of DDT itself. To date, such predictions of pre-selected resistance have been associated with *kdr* alleles of the *para*-encoded sodium channel target, which also show cross-resistance to both DDT and certain pyrethroids. Third, faced with the difficulty of genetically transforming their non-model African mosquito, the group used the mosquito gene to make a resistant-GSTe2 (119 F)-carrying transgenic strain of *Drosophila*. This approach nicely sidesteps the difficulties of re-inventing transgenesis in the insect under study while still conserving the necessary cofactors and physiological environment required for functional recombinant GST expression in insects themselves. Lo and behold, the transgenic flies were indeed resistant to a discriminating dose of DDT, whereas their counterparts lacking the genetic machinery to overexpress GSTe2 were not - proving that expression of this GST alone is sufficient to cause DDT resistance.

Finally, they determined the three-dimensional structure of both the mutant and the wild-type enzyme in order to make predictions about how the resistance-associated amino acid replacement alters the active site of the enzyme. The GSTe2 enzyme structure comprises two domains (termed the N- and C-terminal) connected by a short hinge-like loop or linker, each domain containing bundles of α-helices (termed H1 to H3 in the N-terminal domain and H4 to H8 in the C-terminal domain). The active site of the enzyme, where DDT would have to reside to be chemically attacked, is located deep in a cleft at the interface between the two domains (formed by the interaction of H1 and H3, and H4, H6 and H8). The L119F amino acid replacement is within helix H4, and its overall effect is to open the active site cleft through movement of both H4 and H8 together. The active site of GSTe2 can be divided into two sub-sites. Sub-site one is called the ‘G-site’, where the reduced glutathione is bound to the chemical being attacked. Sub-site two is the ‘H-site’, which recognizes the hydrophobic substrate, in this case DDT. The Leu119 residue normally forms part of the solvent-inaccessible hydrophobic core of the enzyme. However, to accommodate the bulkier side chain of the replacement Phe119, the end of H4 undergoes an impressive bend that, in turn, increases the size of the H-site, thus increasing the size and altering the shape of the site available for docking to DDT. Unfortunately, as it was not possible to crystallize the resistant GST protein as a complex with DDT itself, it remains a little uncertain as to exactly how this altered binding pocket catalyzes increased turnover of DDT. However, comparative analyses with other mosquito GST proteins, and taking into account the known metabolic activities of these proteins against DDT, support the hypothesis that the active site of the resistant GSTe2 enzyme better accommodates DDT in a close-to-reactive conformation.

## Population genetics and insecticide usage

Following this path of investigation from resistant strain to candidate enzyme and putative resistance-associated mutation, the authors also used population genetics to support their case that L119F is indeed the causal mutation for DDT resistance in GSTe2. Thus, if it is the case that the L119F allele has been under strong selection from the past widespread use of DDT, one might expect to see reduced *GSTe2* haplotype diversity in the Pahou population relative to other African populations. This is indeed what the authors found, with only two haplotypes being present in the Benin mosquitoes, in contrast to seven to ten haplotypes in those from other countries. Similar reduced genetic diversity around resistance-associated mutations has been inferred for the *Drosophila* DDT-resistance-associated P450 gene *Cyp6g1*. Here, the footprint of an *Accord* retrotransposon has caused upregulation of a P450 gene product capable of metabolizing DDT [5]. However, later work has in fact shown that several subsequent resistant alleles have evolved, involving both duplication of the *Cyp6g1* locus and the insertion of further transposons [6]. It will therefore be interesting to see whether the changes in GSTe2 of *A. funestus* are followed by a similar adaptive walk, in which new mutations are thought to increase resistance to DDT but decrease the fitness costs incurred in the absence of pesticide.

Interestingly, the distribution of the L119F *GSTe2* haplotype in Africa is strikingly similar to the distribution of the A296S *Rdl* haplotype, which confers resistance to dieldrin [7], probably reflecting similar patterns of gene flow for the two resistance alleles. The A296S replacement in the *Rdl*-encoded γ-aminobutyric acid receptor subunit adds a single hydroxyl group to the alanine side chain in a crucial position in the receptor that both changes drug binding and also allosterically reduces the amount of time that the receptor spends in the drug-preferred ‘desensitized’ state [8]. This similarity in the population genetics of the DDT-resistance-conferring allele of *GSTe2* and the dieldrin-resistance-conferring allele of *Rdl* means that they are both absent from South Africa, consistent with the continued susceptibility of South African mosquito populations to both chlorinated hydrocarbons. This has led to the interesting paradox that both of these aging compounds might yet again prove useful in mosquito control, despite long-term concerns about their environmental effects.

## Implications for the study of resistance

In one fell swoop, Riveron and colleagues have therefore dramatically raised the bar for future resistance studies, which now clearly need to bring all aspects of genomics, biochemistry, structural biology and population genetics to bear on any given resistance gene. It is therefore now worth stopping to reflect on how such interdisciplinary studies can be more readily achieved in this age of genomics and transcriptomics. In times gone by, researchers were forced to put their best postdoctoral research fellow onto the task of cloning their favorite resistance gene from any given non-model insect. In the absence of a genome or indeed a transcriptome from a tissue of interest, this would often involve a considerable amount of guesswork as to what the resistance gene might encode - a target site or metabolic enzyme - and then, most often, the use of degenerate primers in the PCR reaction to try and clone the gene thought to be the most likely candidate. If a fragment of the candidate gene was cloned, a full-length clone then needed to be fished out of a high-quality cDNA library and functional expression studies attempted to prove that the enzyme or receptor did indeed interact with the pesticide in question. Now, the starting point for any work in a non-model organism is a high-quality transcriptome from the whole animal or a tissue of interest (the gut or fat body in insects). This transcriptome can be readily searched for genes encoding likely receptors or enzymes, and these can then be used to inform expression analyses (performed using RNA-seq, deepSAGE or even microarrays) comparing a range of resistant and susceptible strains.

This major transformation in the way we do resistance research has meant that candidate resistance genes can now be identified extremely rapidly, often in insects with little or no genetics [9,10]. Of course, there will still be a need to follow up candidate resistance genes with heterologous expression, insecticide binding and population-genetic studies, but the age of genomics and transcriptomics now allows researchers to be one step closer to any candidate gene in any given insect.

## Abbreviations

DDT: Dichlorodiphenyltrichloroethane; GST: Glutathione S-transferase; kdr: Knockdown resistance; para: Paralytic; RT-PCR: Reverse transcriptase-polymerase chain reaction; Rdl: Resistance to dieldrin.

## Competing interests

The author declares that he has no competing interests.
